# Supplementary figures and images for: Systematic identification of genes encoding cell surface and secreted proteins that are essential for in vitro growth and infection in Leishmania donovani
Source: PLoS Pathog. 2022 Feb 24;18(2):e1010364. doi: 10.1371/journal.ppat.1010364 (PMC8903277; doi:10.1371/journal.ppat.1010364)

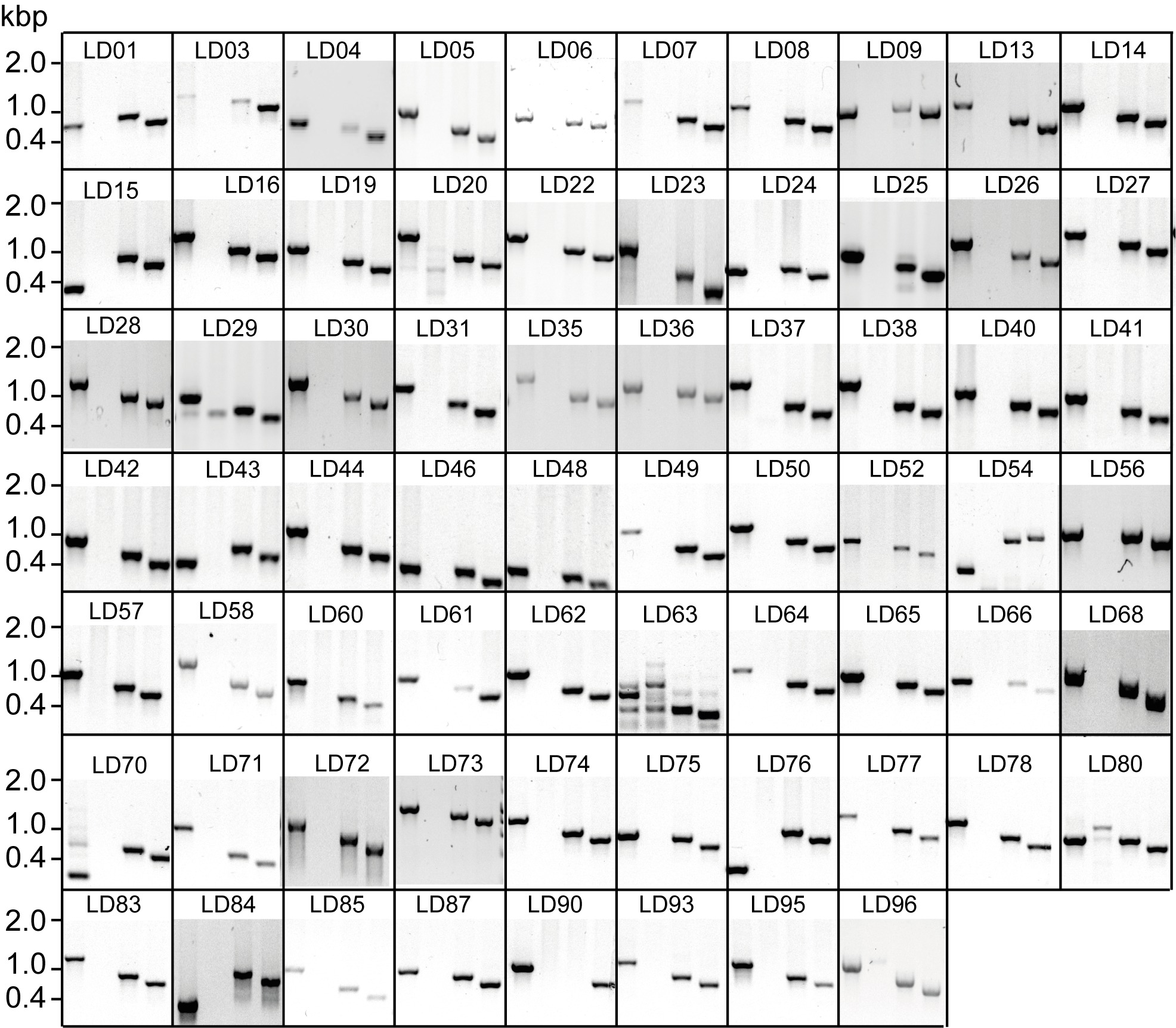

Supplement: S1 Fig — Individual boxes show diagnostic PCR products amplified from genomic DNA extracted from parasites resistant to both puromycin and blasticidin to confirm correct gene targeting; each gene is indicated using the systematic numbering system. For each gene, the products of four PCRs are shown. Lanes 1 and 2 indicate presence of the native allele in genomic DNA extracted from the parental L. donovani strain (lane 1), and targeted doubly-drug resistant parasites (lane 2). Locus-specific targeting with each of the drug resistance genes was demonstrated using primers that were specific to the target locus and either the puromycin (lane 3) or blasticidin (lane 4) resistance cassettes. Note that for gene LD54, the lanes that the PCR products using the puromycin and blasticidin-specific primers were switched. Multiple off-target amplification bands were detected in the LD63 amplifications, and a slower migrating band was observed in the LD80 null mutant. The diagnostic amplification of the correct integration of PAC into the LD90 locus failed on multiple occasions, but we deemed it to be a null mutant as the parasites were resistant to puromycin and the amplification of the endogenous locus showed disruption. (TIF) [file ppat.1010364.s006.tif]

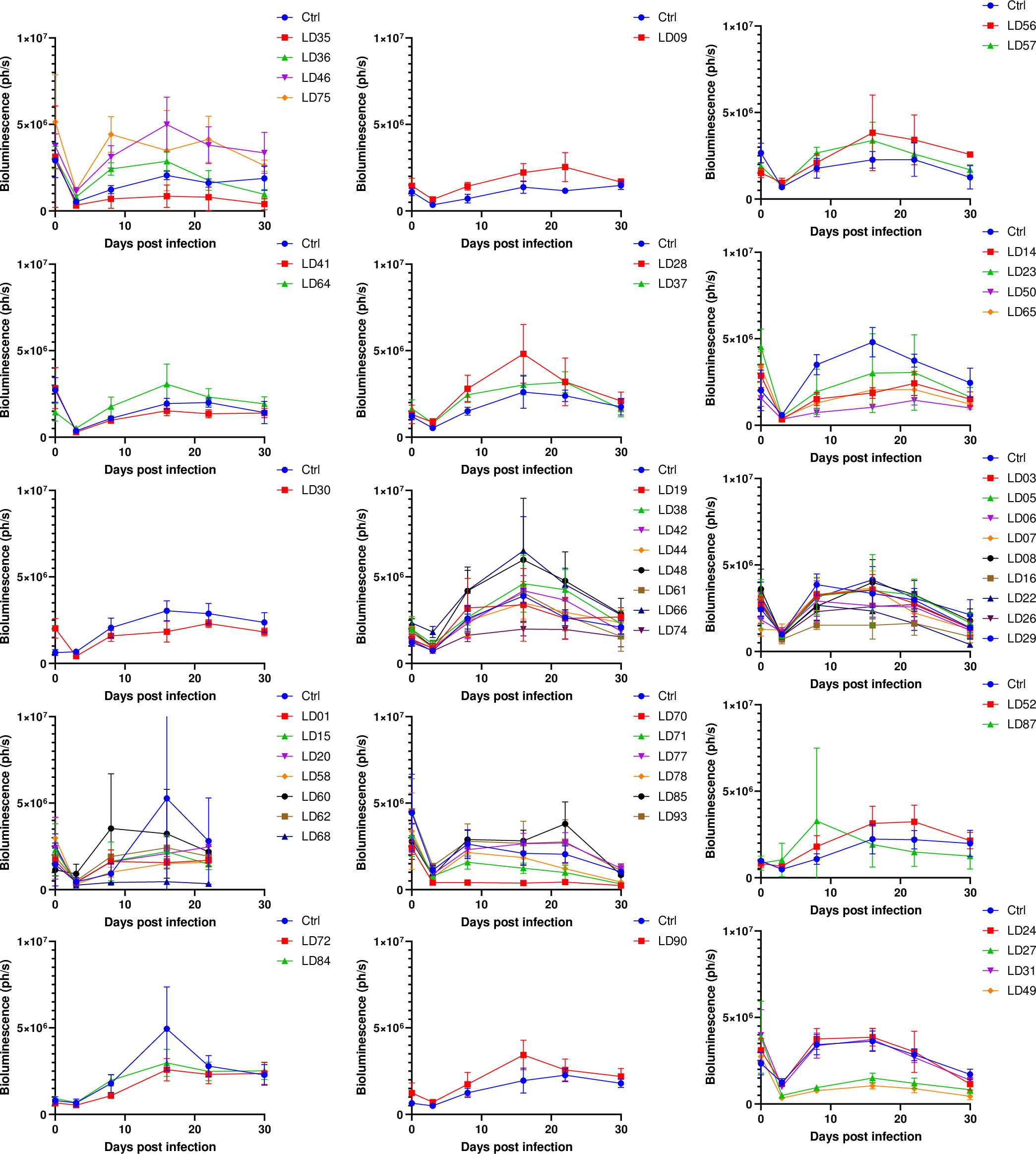

Supplement: S2 Fig — Parasitemia in groups of five female BALB/c mice that had been challenged with 1 x 10^8^ stationary phase gene-targeted L. donovani promastigotes was quantified using bioluminescent imaging at days 0, 3, 8, 16, 22 and 30 days post infection. Separate graphs plot the results from cohorts of mice infected with the indicated mutant parasites. Data points represent means ± s.d.; n = 5. (TIF) [file ppat.1010364.s007.tif]
