# Supplementary material for: Systematic identification of genes encoding cell surface and secreted proteins that are essential for in vitro growth and infection in Leishmania donovani
Source: PLoS Pathog. 2022 Feb 24;18(2):e1010364. doi: 10.1371/journal.ppat.1010364 (PMC8903277; doi:10.1371/journal.ppat.1010364)
Supplement: Supplementary file 7 [file ppat.1010364.s008.docx]

The 5’gRNA for Ld04 gene was designed with the wrong orientation in the manuscript:

gaaattaatacgactcactataggTTGTTGGGCGCAGAGACCAAgttttagagctagaaatagc X

The correct 5’gRNA design for Ld04 gene should be as follows:

gaaattaatacgactcactataggTTGGTCTTTGCGCCCAACAAgttttagagctagaaatagc

This explains why the authors were unable to get any antibiotic resistant cells after six repeated transfections even with the presence of episomal copy of this Ld04 gene.
