# Supplementary material for: Systematic identification of genes encoding cell surface and secreted proteins that are essential for in vitro growth and infection in Leishmania donovani
Source: PLoS Pathog. 2022 Feb 24;18(2):e1010364. doi: 10.1371/journal.ppat.1010364 (PMC8903277; doi:10.1371/journal.ppat.1010364)
Supplement: Attachment — Submitted filename: Roberts_PLoS_Pathogens_rebuttal.docx [file ppat.1010364.s009.docx]

We thank the reviewers for their valuable time spent reviewing our manuscript. We were pleased with the positive tone of the reviews and we have revised our manuscript based on their comments as detailed in our point-by-point response below.

***Part I - Summary*** *Please use this section to discuss strengths/weaknesses of study, novelty/significance, general execution and scholarship.*

*Reviewer #1: The study by Roberts et el represents a considerable body of work to target 92 genes encoding secreted or membrane bound proteins, with the rational that antibodies against some of these secreted proteins may impair parasite survival and thus may represent vaccine targets. The rational for this approach is interesting, testable as conducted in this study and has the study therefore has strong merit. One consideration however is that although the Leishmania protein may be secreted, it may not make it outside the phagolysosome or the infected macrophage and thus would still not be accessible to extracellular antibodies. Nevertheless, I believe the determining the role of this interesting set of leishmania genes in parasite survival in mice is an important study. What is perhaps unexpected was how few of these 92 genes are actually needed for parasite survival in the mouse (none!) although some led to attenuation. Targeting these gene products with antibodies was an interesting approach.*

We thank the reviewer for their positive assessment of this project. The reviewer is correct in their assertion that these proteins may not make it outside of the infected macrophage as there is very limited information on circulating biomarkers during *Leishmania* infections and this was one of the main reasons why we opted for the systematic reverse genetics/vaccinology approach we used in this study.

*Reviewer #2: In spite of the flawless execution of the study, the fact remains that the majority of the work gave inconclusive results. None of the preselected genes have a conditional effect during the mammalian stage of the parasite. There are some marginal growth effects, but no critical impact on the infectivity/pathogenicity of the parasites. Immunization of mice with two selected proteins (LD04, LD11) did not protect against an initial burst of parasite burden in the liver, but prevented the increase of parasite burden in the spleens of the animals, indicating a protective effect against chronic, visceral infection. However, the use of a Students t-test with a sample size of n=5 is not ideal; a non-parametric test (ANOVA, U-test) might be more appropriate. The results should also be shown as a scatter graph to give an idea of the variances.*

We thank the referee for their comments. We explored the referee’s suggestion of showing the individual data points as a scatter graph but found that for most of the time points, the variances between individuals were minimal and therefore made the graph cluttered and difficult to read.

We have taken the referee’s advice and performed a non-parametric Mann-Whitney U-test and the results are tabulated below.

|  |  | **Spleen** | |  |  |  |  | **Liver** | |  |
| --- | --- | --- | --- | --- | --- | --- | --- | --- | --- | --- |
|  | **LD04** |  | **LD11** |  |  |  | **LD04** |  | **LD11** |  |
| Week |  |  |  |  |  | Week |  |  |  |  |
| 1 | 0.7717 |  | 0.3945 |  |  | 1 | 0.0209 | * | 0.1915 | ns |
| 2 | 0.613 |  | 0.319 |  |  | 2 | 0.6132 |  | 0.8518 |  |
| 3 | 0.0565 |  | 0.0019 |  |  | 3 | 0.5028 |  | 0.7068 |  |
| 7 | 0.4325 |  | 0.1264 |  |  | 7 | 0.2787 |  | 0.431 |  |
| 9 | 0.0195 | * | 0.0071 | ** |  | 9 | 0.0701 |  | 0.0708 |  |
| 11 | 0.0482 | * | 0.017 | * |  | 11 | 0.4879 |  | 0.2034 |  |
| 17 | 0.0125 | * | 0.0089 | ** |  | 17 | 0.0011 | ** | 0.0008 | *** |
| 20 | 0.0075 | ** | 0.0208 | * |  | 20 | 0.102 |  | 0.0627 |  |

The outcome of this test are almost identical with the t-test. They both show that there are statistically significant differences between the LD04 and LD11 vaccinated animals and control group in the spleen at weeks 9, 11, 17 and 20. This statistical test did additionally show a significant protective effect in the liver with both antigens at 17 weeks but we would question the biological relevance of this because the infection has largely resolved from the liver by week 6.

*Reviewer #3: In this manuscript, Roberts et al describe a study that uses CRISPR/Cas9 to systematically target 92 genes from Leishmania donovani encoding proteins that are predicted to be secreted or anchored to the external cell membrane. Only 5 genes appeared to be essential for in vitro growth of promastigotes, although they were also unable to obtain null mutants for another 12. Nine additional genes appeared to be important for virulence in the mammalian host, since null mutants showed attenuated infection in mice. Finally, the proteins encoded by the 5 essential genes were used to vaccinate mice and two showed significant protection against splenic infection. The study is well designed with adequate controls and, in general, the manuscript onlis well-written and the conclusion (that the two proteins should be further investigated as vaccine candidates) is warranted.*

We thank the referee for their comments and the time taken to review our manuscript.

***Part II – Major Issues: Key Experiments Required for Acceptance*** *Please use this section to detail the key new experiments or modifications of existing experiments that should be absolutely required to validate study conclusions.

Generally, there should be no more than 3 such required experiments or major modifications for a "Major Revision" recommendation. If more than 3 experiments are necessary to validate the study conclusions, then you are encouraged to recommend "Reject".*

*Reviewer #1: There is no major issue with the study other than the obvious; the strategy failed to identify a vaccine target. However, I applaud the effort since there was some rational in the approach and it was very interesting to learn that none of the targeted proteins are needed for survival in the mouse model.*

We thank the reviewer for their comments.

*Reviewer #2: Given the difficulties in producing the vaccination proteins, the use of recombinant virus vaccination should be considered to broaden the last part of the study.*

This is an interesting suggestion from the reviewer and one that will be seriously considered in our future research. That said, it is not immediately clear to us how using a viral delivery system would lead to improvements in demonstrable protein expression. We understand that a recombinant virus will use the host cells to produce the protein, which is essentially what we are trying to achieve by transfecting human HEK293 cells with the subunit vaccine expression plasmids. In our experience, we find that around 20 to 25% of parasite proteins are not secreted by HEK293 cells – a number that is consistent between different parasites including *Plasmodium* spp., *Schistosoma mansoni, Babesia microti* and *Leishmania donovani*. Although we don’t have direct evidence, we suspect that the failure to express some proteins is more likely to be due to different biological contexts: factors that are present in the parasite that are necessary for successful protein secretion. In these instances, we usually find that the protein is translated (found in the cell pellet), but not secreted and therefore is likely to be recognised by the unfolded protein response and degraded before it is secreted. We would argue that expressing the protein using a recombinant virus would result in the same problem. As the reviewer is perhaps suggesting, recombinant viruses may be effective at eliciting cell-mediated immunity even if the protein isn’t secreted. While this could be an attractive strategy for developing a vaccine, our initial rationale was to elicit humoral immunity by targeting cell surface and secreted proteins.

*Reviewer #3: There are no major issues that need to be addressed.*

Thank you.

*Part III – Minor Issues: Editorial and Data Presentation Modifications
Please use this section for editorial suggestions as well as relatively minor modifications of existing data that would enhance clarity.*

*Reviewer #1: I had a number of comments which I would place in the Minor issue category as listed here.

Why were the genes needed for promastigote growth selected for vaccine targets, I would have thought the genes needed for optimum survival in the mouse would have been equally good targets? Examples Ld68, LD70, Ld50*

We agree with the reviewer that these candidates may be good vaccine targets given the reduced virulence of the gene-deficient parasites in mice. In this study, we have opted for those that were essential in the promastigote stage under the premise that the parasites cannot survive without their presence.

*With respect to CL parasites protecting against VL parasites (line74-75), a newer reference should now also be included (*[*https://doi.org/10.1038/s42003-021-02446-x*](https://doi.org/10.1038/s42003-021-02446-x)*)*

We would like to thank the reviewer for this suggestion of which we have now included in our revised manuscript (line 75, reference 12).

*The first results paragraph describes the development of the transgenic line, how it was unclear from the description how the luciferase gene was expressed in this line.*

The selection of the luciferase-expressing transgenic parasite line was been published (Ong *et al*. “Establishment, optimisation and quantitation of a bioluminescent murine infection model of visceral leishmaniasis for systematic vaccine screening” Sci Rep. 2021 Apr 7;11(1):8015. PMID: 33828148) where these details are available. We have cited this manuscript at the appropriate place in the manuscript.

*Line 179, please provide some explanation how genes in chrom 26 and 31 were targeted using 2 selectable markers for trisomy and tetrasomy chromosomes. Did the same marker enter 2 chromosomes. If so, presumable a single marker could have been used for all the targeted genes and this could have been verified by PCR such as in Table S1.*

We have provided additional text in our revised manuscript to explain a possible mechanism by which three copies of a gene may be targeted by only two selectable markers (line 179-183). As the reviewer suggests, while it is technically possible to generate null mutants using only a single selectable marker, this would almost certainly lead to a higher rate of false null mutants and require more extensive screening of individual clones.

*The description of proliferation in Fig 3A are specific to promastigotes; not parasites since parasites could include amastigotes.*

Thank you – now corrected.

*Line 274; it would have been better to isolate equivalent numbers of metacyclic promastigotes from each culture to perform the BALB/c mouse infections. Mutants with lower levels of infection could have had fewer metacyclics, particularly mutants in membrane proteins.*

Yes, we agree with the reviewer that using an equivalent number of metacyclic promastigates would be a preferable way to directly compare the infection profiles of the different mutants. We invested a lot of effort in optimising our murine infection protocol with this bioluminescent parasite line to reduce variability for our vaccine screening studies, and this work has been published – see: Ong *et al*. “Establishment, optimisation and quantitation of a bioluminescent murine infection model of visceral leishmaniasis for systematic vaccine screening” Sci Rep. 2021 Apr 7;11(1):8015. PMID: 33828148. In our experience, however, the purification of metacyclic promastigotes using either lectin or density purification methods did not result in increased infection signals *in vivo*, and indeed, we found that these methods of enriching for metacyclic promastigotes can introduce an additional layer of variability which makes comparisons between different strains difficult. On balance, we opted to use the whole population of stationary phase parasites as the best comparative method. As the reviewer points out, any differences in the infection phenotype could be due to alterations in the metacyclic differentiation pathway, but as we report in our manuscript, we did not detect any major differences in virulence between the different gene-deficient parasites either *in vitro* or *in vivo*.

*Please see attachment for further comments. [the reviewer provided a document showing that the design for one of our targeting primers (gene ref LD04) was incorrect]*

We would like to thank reviewer one for spotting the error in one of the sgRNA targeting primers used to target the gene referred to as LD04. We have repeated the knockout attempts of LD04 using the correct primer and we have subsequently found this gene be dispensable for growth *in vitro*. We have therefore altered the tables and figures to reflect these results (see revised Fig S1 and Fig 2). We have also made text changes to the manuscript to explain the inclusion of LD04 in the vaccination screen (Lines 315-317).

*Reviewer #2: Everything was concisely presented.*

Thank you.

*Reviewer #3: Lines 75 and 84: there are several instances throughout the manuscript where Leishmania is not italicized.*

Thank you; these have now been corrected in our revised manuscript.

*Line 115: What strain/isolate of L. donovani was used. Isolates from different geographical location show considerable genetic variation and it is important to know precisely which one was used.*

The strain of *L. donovani* used in this study was LV9. This information has now been included in our revised manuscript.

*Line 166: remove the space before the period.*

Thank you – now corrected.

*Line 181: While the identify of all 92 genes (or at least their orthologues in the LdBPK282A1 reference strain) are identified in supplementary Table S2, it might be useful for the reader if a table were added to the main text that summarized at least the 5 essential genes.*

Agreed. We have now included a new main table (Table 1) in our revised manuscript which provides the details of the essential genes identified in this study.

*Figure 3: Replace “systemic” with “systematic”*

Thank you – now corrected

*Lines 402-406: It is not clear what is the point of the discussion about P. berghei genes.*

The reference to the *P. berghei* study was a discussion point on the percentage of genes required for viability in a protozoan parasite. It was found in a major study (see Bushell *et al*. “Functional Profiling of a Plasmodium Genome Reveals an Abundance of Essential Genes. Cell. 2017 170(2) p260-272 PMID: 28708996) targeting genes in *P. berghei* that a large proportion of genes were essential, much higher than the number we have found to be essential in *Leishmania*. We propose that this is due to cell surface proteins being more likely to be required for host interaction and thus be dispensable for growth.

Additional changes not detailed above:

We have included the diagnostic PCR for LD04 following the correction of the primer sequences into Figure S1 and Figure 2D has been altered to remove the LD04 knockout attempts in our revised manuscript.
